# Probing the global and local dynamics of aminoacyl-tRNA synthetases using all-atom and coarse-grained simulations

**DOI:** 10.1007/s00894-014-2245-1

**Published:** 2014-05-09

**Authors:** Alexander M. Strom, Samuel C. Fehling, Sudeep Bhattacharyya, Sanchita Hati

**Affiliations:** Department of Chemistry, University of Wisconsin-Eau Claire, Eau Claire, WI 54702 USA

**Keywords:** Aminoacyl-tRNA synthetase, Coarse-grained simulations, Intrinsic global dynamics, Molecular dynamics, Normal model analysis, Protein dynamics

## Abstract

Coarse-grained simulations have emerged as invaluable tools for studying conformational changes in biomolecules. To evaluate the effectiveness of computationally inexpensive coarse-grained models in studying global and local dynamics of large protein systems like aminoacyl-tRNA synthetases, we have performed coarse-grained normal mode analysis, as well as principle component analysis on trajectories of all-atom and coarse-grained molecular dynamics simulations for three aminoacyl-tRNA synthetases—*Escherichia coli* methionyl-tRNA synthetase, *Thermus thermophilus* leucyl-tRNA synthetase, and *Enterococcus faecium* prolyl-tRNA synthetase. In the present study, comparison of predicted dynamics based on *B*-factor and overlap calculations revealed that coarse-grained methods are comparable to the all-atom simulations in depicting the intrinsic global dynamics of the three enzymes. However, the principal component analyses of the motions obtained from the all-atom molecular dynamics simulations provide a superior description of the local fluctuations of these enzymes. In particular, the all-atom model was able to capture the functionally relevant substrate-induced dynamical changes in prolyl-tRNA synthetase. The alteration in the coupled dynamics between the catalytically important proline-binding loop and its neighboring structural elements due to substrate binding has been characterized and reported for the first time. Taken together, the study portrays comparable and contrasting situations in studying the functional dynamics of large multi-domain aminoacyl-tRNA synthetases using coarse-grained and all-atom simulation methods.

**Electronic supplementary material:**

The online version of this article (doi:10.1007/s00894-014-2245-1) contains supplementary material, which is available to authorized users.

**Figure:**
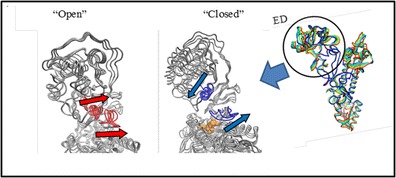
Substrate-induced conformational change in *E. facium* prolyl-tRNA synthetase

## Introduction

**Fig. 1:**
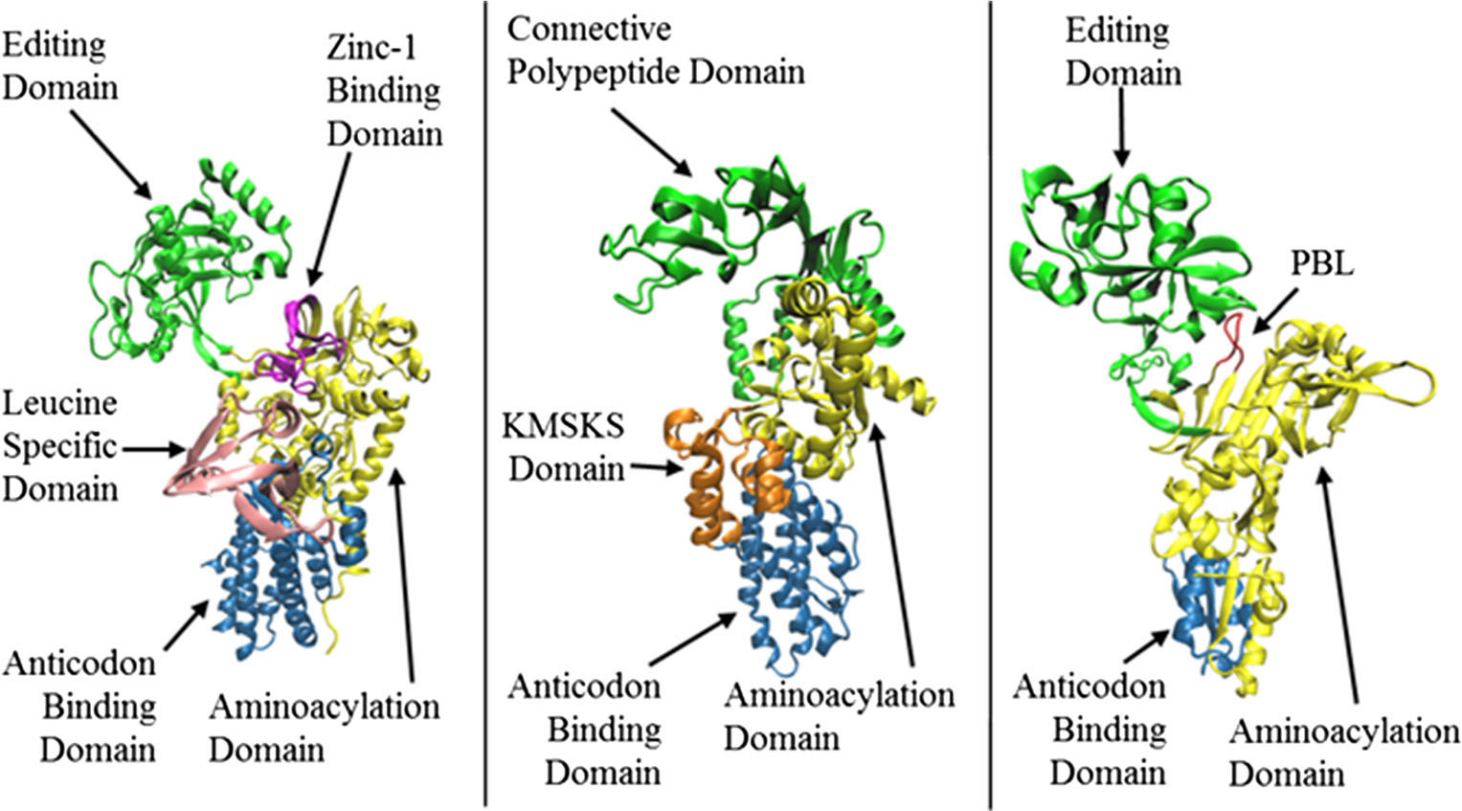
Cartoon representation of the 3D structure of the monomeric form of **a** Tt LeuRS (pdb code: 1H3N, residues 1–814); **b** Ec MetRS (pdb code: 1qqt, residues 3–548); **c** Ef ProRS (pdb code: 2J3M, residues 19–567). The structural domains are colored as follows: **a** *green*, the editing domain (ED; residues 224–417); *purple*, zinc-1 binding domain (ZBD; residues 154–189); *yellow*, aminoacylation domain (residues 1–153, 190–223 and 638–642); *pink*, leucine-specific domain (LSD; residues 577–634); and *blue*, the anticodon binding domain (residues 635–814); **b** *yellow*, aminoacylation domain (residues 1–96, 252–323); *green*, connective polypeptide (CP) domain (residues 97–251); *orange*, KMSKS domain (324–384 and 536–547), and *blue*, the anticodon binding domain (residues 385–535); **c** *green*, editing domain (residues 224–407), *red*, the proline-binding loop (residues, 199–206); *red*, aminoacylation domain (residues 1–223, 408–505), and *blue*, the anticodon binding domain (residues 506–567)

Aminoacyl-tRNA synthetases (AARSs) play a pivotal role in cellular protein synthesis and viability [1]. AARSs are responsible for accurately attaching an amino acid onto the corresponding tRNA molecule in a two-step reaction. The amino acid is first activated with ATP, forming an aminoacyl-adenylate intermediate. The activated amino acid is then transferred to the 3′-end of its corresponding tRNA molecule to be used for protein synthesis. The structure of an AARS is usually composed of a central catalytic core (aminoacylation domain) and the anticodon binding domain (Fig. 1). The central aminoacylation domain is responsible for the selection and activation of the correct amino acid. The anticodon binding domain is often responsible for selecting the corresponding tRNA molecule. In the course of evolution, additional domains were either appended or inserted to the two-domain structure to enhance catalytic efficiency and confer tRNA selection [1]. In some cases, these appended domains exclusively catalyze editing reactions by hydrolyzing the misactivated amino acids (pre-transfer editing reaction) and/or misaminoacylated tRNA (post-transfer editing reaction) [2].

These multi-domain enzymes undergo large conformational changes. In particular, it has been observed that substrate binding and aminoacyl-adenylate formation triggers local conformational changes, which are coupled with global dynamics of distant structural elements [3, 4]. Also, during post-transfer editing reaction, the misacylated tRNA has to translocate to the editing domain from the aminoacylation domain and the large-scale conformational change of the editing domain has been observed in the tRNA-bound crystal structures of AARSs [5]. Recent studies from our group have demonstrated that coupled-domain dynamics play important roles in AARSs’ functions [4, 6, 7]. However, it is not clearly understood how these global dynamics are relevant for local conformational changes that aid in substrate binding and product release and catalysis in these enzymes.

One of the important areas in enzymology in recent years is the role of protein dynamics in enzyme catalysis. Recent studies have suggested that the coupling between intrinsic global and local dynamics of a protein play an important role in substrate recognition and binding [8]. However, the role of intrinsic dynamics on enzyme catalysis is shrouded in mystery [9–19]. It has been proposed that “coupling between the catalytic site and collective dynamics is a prerequisite for mechanochemical activity of enzymes” [17]. In particular, it has been proposed that these collective dynamics can modify the catalytic rate by influencing the height of the activation free energy barrier and the transmission coefficient (i.e., the capacity of recrossing the barrier) [12, 18]. Studies by other groups suggested that the collective dynamics is significant to promote a pre-organized active site conformation that is critical for catalysis to happen [19]. Therefore, in order to obtain an insight of the interplay of dynamics and function of an enzyme like AARS, it is critical to study both local conformational and global dynamical changes.

Capturing biologically relevant conformational transitions in large protein systems requires long-timescale MD simulations, which are computationally intensive processes. On the other hand, the computationally inexpensive approaches such as coarse-grained MD simulation (CMD) [20] and normal mode analysis (NMA) [21, 22] could be equally useful to study large-scale conformational changes. Despite the limitations of harmonic approximations in describing large-amplitude displacements [21], normal mode analysis has been routinely used for analyzing domain motions [23]. In a recent study Bahar et al. have shown that the coarse-grained model is able to mimic global motions occurring in the time scale of millisecond [24]. Separately, using coarse-grained and all-atom simulations of human tyrosyl-tRNA synthetase, Yesylevskyy et al. have obtained important information about inter-domain interactions [25, 26]. Although coarse-grained simulations methods can capture global motions appropriately, it is not always informative about the local interactions and its role in shaping global dynamics. In the present study, an effort has been made to compare and contrast the motional (global and local) information obtained from the coarse-grained and all-atom simulations for three AARS systems, namely, *Thermus thermophilus* leucyl-tRNA synthetase (Tt LeuRS), *Escherichia coli* methionyl-tRNA synthetase (Ec MetRS), and *Enterococcus faecalis* prolyl-tRNA synthetase (Ef ProRS). These three enzymes are structurally and biochemically well characterized and have a highly flexible domain inserted into the relatively rigid central aminoacylation core domain (Fig. **1**).

## Methods

Starting X-ray crystallographic protein structures were taken from Protein Data Bank (PDB) [27]. Specifically, PDB codes 1H3N for Tt LeuRS (residues 1–814) [28], 1QQT for Ec MetRS (residues 3–548) [29], and 2J3M for Ef ProRS (chain B, residues 19–565) [3] were used for simulations. The substrate-bound structure was obtained by docking the prolyl-adenylate into the catalytic (aminoacylation) domain of Ef ProRS using Argus lab (http://www.arguslab.com/ arguslab.com/ArgusLab.html).

### Molecular dynamics simulations

The details of the MD simulation protocol were described previously [4, 7]. Briefly, for all simulations, the all-atom CHARMM22 force field [30] was used within the NAMD [31] package. The three-point charge TIP3P model [32] was used to represent solvent water. Non-bonded interactions were truncated using a switching function between 10 and 12 Å, and the dielectric constant was set to unity. The SHAKE algorithm [33] was used to constrain bond lengths and bond angles of water molecules and bonds involving a hydrogen atom. The MD simulations were performed using isothermal-isobaric (NPT) conditions. Periodic boundary conditions and particle-mesh Ewald methods [34] were used to account for the long-range electrostatic interactions. In all molecular dynamics simulations, a time step of 2 fs was used. The pressure of the system was controlled by the implementation of the Berendsen pressure bath coupling [35] as the temperature of the system was slowly increased from 100 to 298 K. During the simulations at 298 K, the pressure was kept constant by applying the Langevin piston method [36, 37]. All simulations were conducted with a 500 ps equilibration step followed by a 30 ns production MD run. Additionally, in the case of Tt LeuRS, a total of 55-ns of simulated data were generated to perform an analysis of the effect of simulation time on the nature of dynamic information. The equilibration and stability of each system was checked by calculating the root-mean-square deviations (RMSD) of C_α_ atoms from their initial coordinates using the simulated data obtained in the production phase (Fig. S1). The RMSD values were observed to fluctuate within 1.0–2.5 Å during the production period, indicating stability for each system.

### Essential dynamics analysis

The collective dynamics of the protein was studied through essential dynamic analysis (EDA) [38–40], which involves computation of the principal components of atomic fluctuations. The details of the procedure have been described earlier [4, 7, 41]. The last 25 ns of the 30 ns MD simulation data was used to extract the principal modes of collective dynamics (called principal components) using the program CARMA [42]. The principal components analysis (PCA) were computed by performing eigenvalue decomposition of a covariance matrix, and the mathematical formulism is described elsewhere [43]. Briefly, PCA was carried out using the following steps: (i) preparing a modified trajectory file by removing the coordinates of the water molecules, selecting only the C_α_ atoms, and removing the overall translational and rotational motions, (ii) calculating the covariance matrix in which the atomic coordinates are the variables, and (iii) diagonalizing the covariance matrix for calculation of the eigenvectors and their corresponding eigenvalues. The first three PCs were used for performing PCA-based cluster analysis as discussed in CARMA documentation [42]. Based on contributions of the first three PCs, conformations along the MD trajectories were grouped into several clusters. The cluster with the greatest number of conformations per unit fluctuation was used for further analysis of dynamic cross-correlations between C_α_ atoms.

### Normal mode analysis

In the present study, normal mode calculations were carried out as described earlier [6]. Briefly, a coarse-grained NMA [22, 44, 45] was used by employing elastic network model, which is described in the next section. In this model, each protein structure is simplified by treating it as a network of pseudo-atoms. These pseudo-atoms are C_α_ atoms connected to the other neighboring C_α_ atoms, within a certain distance cutoff, by springs of uniform force constant [20]. We used the Anisotropic Network Model [46]. The online server, http://ignmtest.ccbb.pitt.edu/cgi-bin/anm/anm1.cgi, was used to obtain the simulated motion for the three protein systems [22]. In the present study, the optimal distance cut-off and distance weight for interactions between C_α_ atoms were kept at 10 and 2.5 Å, respectively [22]. The correlated or anticorrelated motions between residue pairs of distant structural elements were determined from the cross-correlations of residue pairs (for the combined modes 1–3). The cross-correlation coefficient between fluctuations of residues *i* and *j* (*CC*_*ij*_) was calculated using

1 $$ C{C}_{ij}=\frac{\langle \left({x}_i-\langle {x}_i\rangle \right)\left({x}_j-\langle {x}_j\rangle \right)\rangle }{\sigma_{x_i}{\sigma}_{x_j}} $$

where ${\sigma}_{x_i}$ and ${\sigma}_{x_j}$ represent the standard deviation of the displacements of the two points (C_α_ coordinates) *i* and *j*, respectively. The correlated motion (*CC*_*ij*_> 0) between two C_α_ atoms occurs when they move in the same direction while the anticorrelated motion (*CC*_*ij*_ < 0) is generated when two C_α_ atoms move in the opposite direction.

### Coarse-grained molecular dynamics

CMD simulations were conducted using the program Reduced Molecular Dynamics (RedMD) [20]. The biomolecular model was generated using reduced representation of each protein system. In this coarse-grained representation, the generic simple harmonic elastic network model was employed, where each C_α_ atom is represented by a spherical bead with a mass corresponding to the total mass of a given amino acid. The overall potential of the system is expressed as the sum of harmonic potentials between the interacting C_α_ atoms according to Eq. 2:

2 $$ {E}_{\mathrm{ENM}}=\frac{\gamma }{2}\left[{\displaystyle \sum_{j,i\ne j}}{\varGamma}_{ij}\left[{\left({r}_{ij}-{r}_{ij}^{\mathrm{o}}\right)}^2\right]\right]. $$

In Eq. 2, *γ* represents the uniform spring constant, *r*_*ij*_^o^ and *r*_*ij*_ are the original and instantaneous distance vectors between residues *i* and *j*, and *Γ*_*ij*_ is the *ij*th element of the binary connection matrix of inter-residue contacts. Based on an interaction cut-off distance of *r*_*c*_, *Γ*_*ij*_ is equal to 1 if *r*_*ij*_^o^ < *r*_*c*_ and zero otherwise. The uniform force constant of C_α_-C_α_ bonds was set to 1.0 kcal (mol·Å^2^)^−1^ with a cut-off (*r*_*c*_) of 8.0 Å. A total of 10 ns CMD simulations were performed using a 0.02 ps time step. The temperature was maintained at 298 K. A canonical ensemble (NVT) was generated for further analysis. The CMD derived trajectory was analyzed in the same way using essential dynamics analysis as described earlier for the all-atom MD simulation and hereafter will referred as CEDA.

### *B*-factor calculations

In order to quantify thermal motions associated to the collective dynamics of these protein systems, the thermal fluctuations were calculated for various simulation types. The *B*-factors of individual C_α_ atom were extracted from the thermal fluctuation column in the averaged-structure coordinate file obtained for the MD and CMD. Similarly, the NMA thermal fluctuations of all principle modes were obtained from the average structure. Lastly, experimental (X-ray crystallographic) *B*-factors from the PDB files were used for comparison.

### Overlap calculations

The overlap between the conformational change predicted by different simulation methods was calculated using Eq. 3, formulated by Marques and Sanejouand [47],

3 $$ {O}_{Method1- Method2}=\frac{\left|{\displaystyle \sum_i^N}\varDelta {r}_i^{Method1}\times \varDelta {r}_i^{Method2}\right|}{\sqrt{{\displaystyle \sum_i^N}{\left(\varDelta {r}_i^{Method1}\right)}^2\times {\displaystyle \sum_i^N}{\left(\varDelta {r}_i^{Method2}\right)}^2}}, $$

where *Δr*_*i*_^*Method*1^ represents the displacement from the original position of the protein’s C_α_ atoms observed in method 1. According to Eq. 3, an overlap value of 1.0 means that the magnitude of a C_α_ atom displacement predicted by method 1 is identical with the one predicted by method 2.

## Results and discussion

The results of this study consist of the similar and contrasting features between all-atom and coarse-grained simulations and these are presented in the following order. First, in order to evaluate the correspondence between various simulation methods, a comparison of normalized thermal fluctuations produced by each simulation method is reported. Second, the calculated overlap values between different simulation methods for the full-length protein, as well as selected domains of each of the three AARSs have been compared. Third, the correlated/anticorrelated motions between different structural elements have been analyzed and compared between the three simulation methods. Fourth, the correspondence between local and global motions in substrate-bound and substrate-free Ef ProRS system is presented.

### Thermal fluctuations

**Fig. 2:**
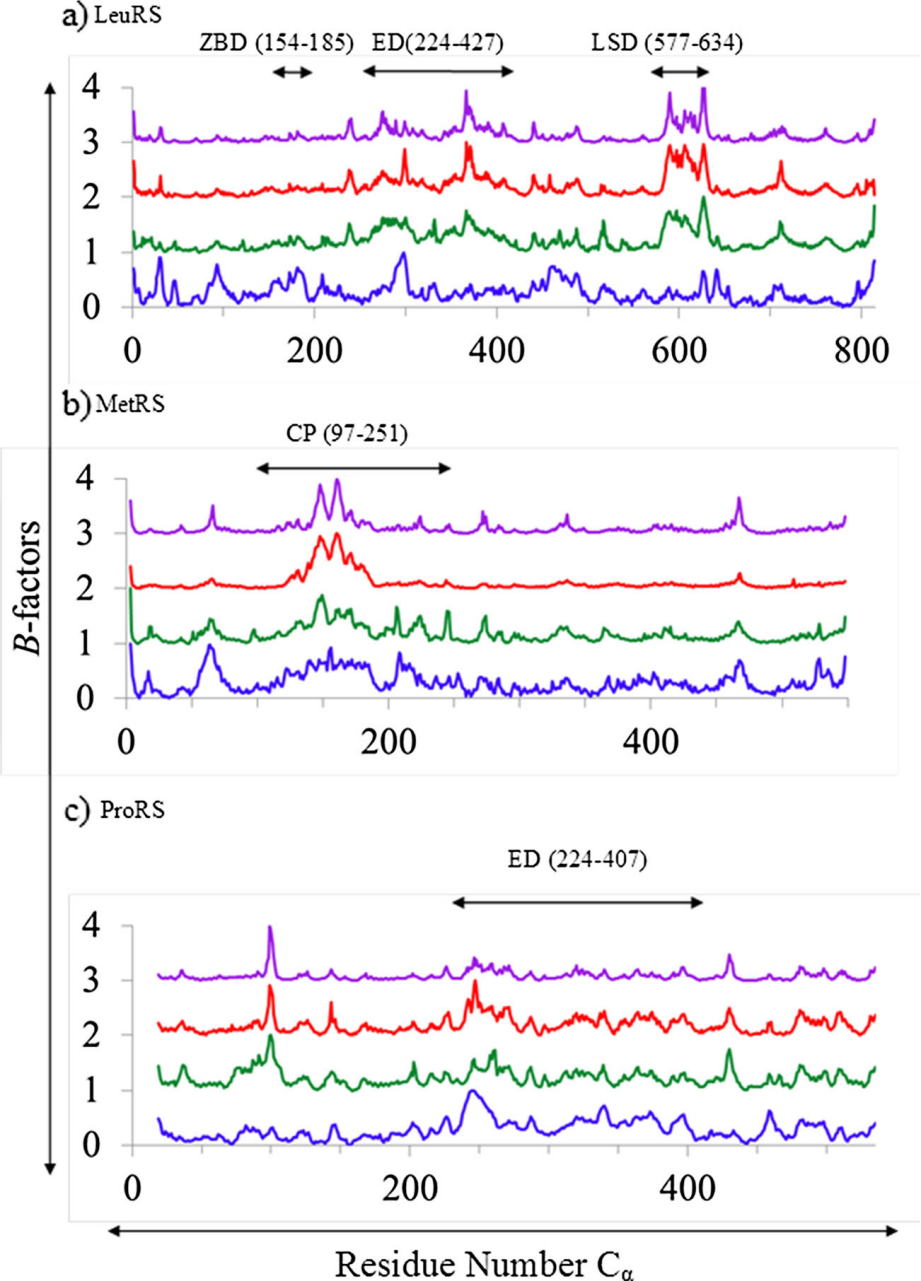
Simulated and normalized *B*-factors produced by crystal structure (*blue*), EDA (*green*), CEDA (*red*), NMA (*purple*), for the three systems: **a** Tt LeuRS, **b** Ec MetRS, **c** Ef ProRS

**Fig. 3:**
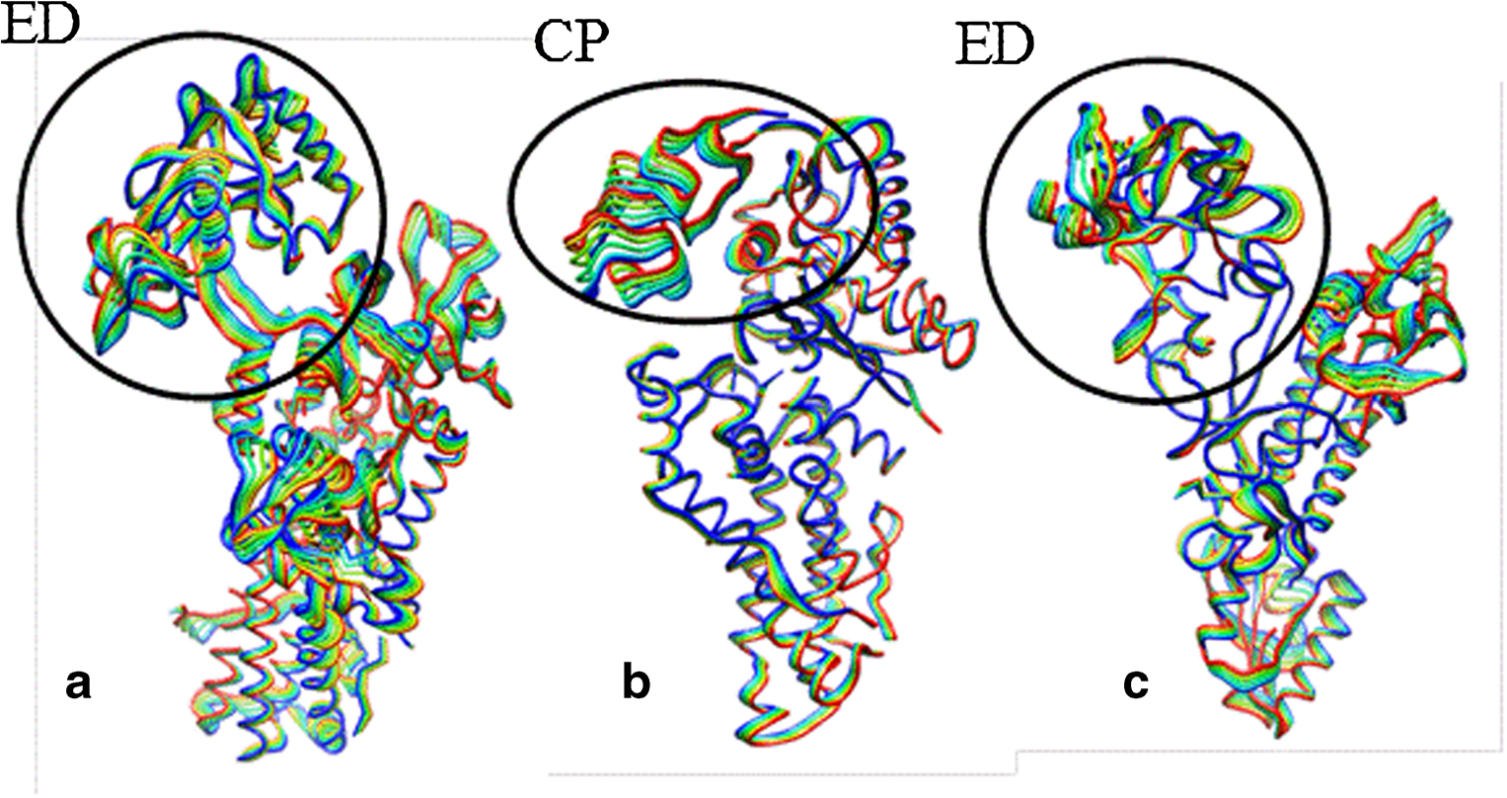
The most flexible domain of each AARS is indicated by a black circle/oval. Each color represents a different conformation obtained from the essential dynamics trajectories of principle component 1 for each enzyme system. **a** Editing domain (ED, residues 224–417) domain of Tt LeuRS, **b** Connective polypeptide domain (CP, residues 97–251) of Ec MetRS, **c** Editing domain (ED, residues 224–407) of Ef ProRS

The *B*-factor analysis was performed for the three AARSs to analyze the backbone flexibility. A plot of normalized experimental (crystallography) and calculated *B*-factors is shown in Fig. 2. The flexible regions identified by EDA, NMA, and CEDA methods are quite comparable for all three enzymes. For example, a similar flexibility pattern was revealed by these methods for the most flexible domain in these enzymes—the editing domain (ED, residues 224–417) of LeuRS, the CP domain (residues 97–251) of MetRS, and the ED (residues 224–407) of ProRS (Fig. 3). For LeuRS and ProRS, the PCA (of MD and CMD trajectories) and the NMA produced almost identical plots. However, the magnitude of the predicted flexibility of MetRS backbone from CMD trajectories was smaller compared to that of the MD. Also, it was noted that the *B*-factor plot derived from crystallographic data of the LeuRS system primarily differing for the first 200 residues showing higher thermal flexibility compared to the computed *B*-factor values. Overall, the backbone flexibility pattern of the three proteins was depicted satisfactorily by both atomistic and coarse-grained simulation methods.

It should be noted that these MD-derived thermal fluctuations were obtained from 30 ns simulations. However, a recent study used 100 ns simulation to model energetically favored domain motions and compactizations in tyrosyl-tRNA synthetase [26]. Therefore, long-duration simulations could provide additional information of the dynamics of these proteins. Also, only one MD trajectory was used for each protein system, as our recent study on ProRS did not reveal significant differences in overall fluctuations at room temperature, while using triplicate simulations [7].

### Overlap between computed displacements

While normalized thermal fluctuations offer a simple yet meaningful illustration of the overall flexibility of these proteins, a quantitative analysis of the overall protein motion was performed by calculating the overlap values using Eq. 3. In this study, the calculated overlap values represent the degree of correlation between the displacements computed by two different computational methods. Overlap values for the full protein (all residues) were calculated for the first three low-frequency modes, modes 1–3 (Table 1) for NMA and PC1-3 for EDA and CEDA. In each case, the coarse-grained results were compared to the conformational change in all-atom simulation-derived EDA, which depicts the atomistic effects on the collective dynamics of a molecule. The computed overlap values for the three AARSs ranged from 0.73 to 0.92 between NMA and EDA and 0.61–0.88 between CEDA and EDA. It is apparent that the NMA was slightly better compared to the CEDA in reproducing the residue fluctuations as predicted by EDA by displaying higher overlap values for the first three low-frequency modes (apart from mode 3 of LeuRS). Overall, these data also suggest that the coarse-grained simulation methods are comparable to all-atom MD simulations in depicting the intrinsic slow dynamics of AARSs.

**Table 1:** Overlap values for the displacements corresponding to the first three lowest frequency modes (mode 1- 3) of the three full-length AARSs as computed between all-atom (EDA) and a coarse-grained (NMA or CEDA) simulation methods

Protein system	Mode	EDA and NMA	EDA and CEDA
*Tt* LeuRS	1	0.80	0.61
	2	0.89	0.88
	3	0.88	0.88
	Average	0.86	0.79
*Ec* MetRS	1	0.92	0.87
	2	0.86	0.78
	3	0.77	0.72
	Average	0.85	0.79
*Ef* ProRS	1	0.81	0.80
	2	0.78	0.78
	3	0.73	0.71
	Average	0.77	0.76

Overlap values between coarse-grained and all-atom simulations in predicting the collective dynamics of a specific domain that contributes to the catalytic function of these proteins were also investigated. In the present study, the overlap values of the highly flexible insertion domain (ED of Tt LeuRS, CP of Ec MetRS, and ED of Ec ProRS) were calculated for each enzyme for modes 1 to 3 (Table 2). Higher overlap values (>0.8) were obtained while comparing the domain displacement predicted by all-atom vs. coarse-grained simulations. The interfacial region between the catalytic and the insertion/CP domain are structurally very heterogeneous in terms of secondary structural elements (Fig. S2). Therefore, the greater overlap values obtained in this study indicates similar inter-domain displacements despite variations in local interactions at domain-domain interfaces. This observation demonstrates that both, all-atom and coarse-grained, methods are similar in modeling the global dynamics in these three AARSs.

**Table 2:** Overlap values for the displacement of the most labile domain of the three AARSs computed between all-atom (EDA) and a coarse-grained (NMA or CEDA) simulation method

Domain	EDA and NMA			EDA and CEDA		
	Mode 1	Mode 2	Mode 3	Mode 1	Mode 2	Mode 3
*Tt* LeuRS						
ED (224–417)	0.86	0.84	0.94	0.90	0.91	0.92
ZBD (154–189)	0.92	0.96	0.97	0.96	0.94	0.84
LSD (577–634)	0.84	0.98	0.93	0.92	0.93	0.85
*Ec* MetRS						
CP (97–251)	0.97	0.86	0.82	0.97	0.83	0.68
*Ef* ProRS						
ED (224–407)	0.80	0.83	0.86	0.83	0.84	0.89

### Correlated and anti-correlated motions

**Fig. 4:**
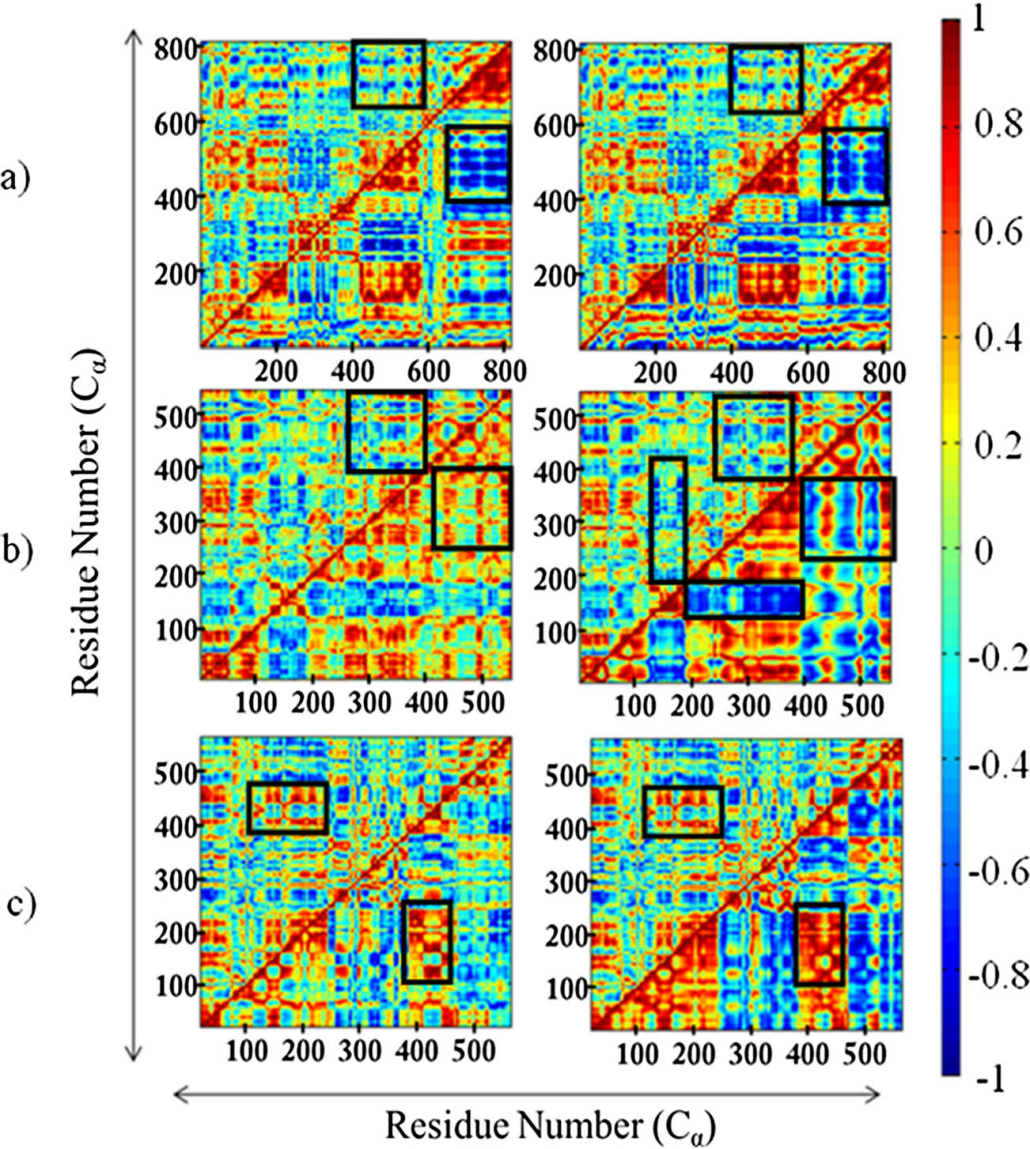
Dynamic cross-correlation map of the three full-length AARSs. **a** Tt LeuRS; **b** Ec MetRS; **c** Ef ProRS. In the left-hand-side matrices, EDA (*above diagonal*) and CEDA (*below diagonal*) and in the right-hand-side matrices, EDA (*above diagonal*) and NMA (*below diagonal*)

Dynamic cross-correlation matrices (DCCMs) were generated using either the first three PCs for EDA/CEDA or the first three lowest-frequency normal modes in the case of NMA (Fig. 4). Analysis of cross-correlations between residue fluctuations revealed both inter- and intra-domain dynamic correlations. As illustrated by the pair-wise comparison (EDA vs. NMA and EDA vs. CEDA) in Fig. 4, the sign of correlations between residue pair fluctuations are very similar when compared between all-atom (EDA) and coarse-grained (CEDA and NMA) simulations for each protein system. Especially, similar patterns of correlation and anti-correlation were observed while considering the relative dynamics of various domains. For example, the central aminoacylation domain [Tt LeuRS, residues 1–153 and 190–223; MetRS, residues 1–96, 252–323, 324–384, and 536–547; and ProRS, residues 1–223, 408–505] and the highly flexible insertion domain [LeuRS, residues 224–417; MetRS, residues 97–251; and ProRS, residues 224–407] are mainly engaged in anticorrelated motions (for all systems); i.e., their displacements are in opposite directions (*C*_*ij*_ < 0). This anticorrelated motion between the ED/CP domain and aminoacylation domain is biologically significant. For example, this observed anticorrelated motion is completely consistent with the structural studies of Tt LeuRS, which shows the ED undergoes a rotation of 35° rotation when complexed with tRNA^Leu^ during the post-transfer-editing reaction. This change in conformation of the editing domain opens up a channel between the aminoacylating (synthetic) and editing active sites facilitating the shuttling of the 3′ terminus of mischarged tRNA^Leu^ from the synthetic active site to the editing active site [6, 48]. The same anticorrelated motion between these domains is also important for other AARSs because this motion allows the corresponding tRNA substrate to access the catalytic site for the aminoacylation reaction.

Similarly, correlated motion (*C*_*ij*_ > 0) between various structural elements within the aminoacylation and the insertion domains were revealed through both coarse-grained and all-atom simulations. However, closer scrutiny of these DCCMs revealed that coarse-grained simulations provided rather imprecise information regarding the residue-residue fluctuations and in contrast, more detailed information were obtained from the MD simulations. These differences between the all-atom and the coarse-grained approaches are discussed below.

#### EDA vs. CEDA

For all three protein systems, it has been found that the intensities of correlation maps obtained from all-atom simulations are relatively low compared to the coarse-grained-derived DCCMs, where the lower-half triangle of the left-hand side matrices corresponds to CEDA (Fig. 4). This observation suggests that the simulated EDA-derived residue fluctuations are less correlated/anticorrelated compared to those obtained using coarse-grained models (Fig. 4). For example, compared to the EDA, the CEDA revealed stronger anticorrelated motions between residues 400–600 (part of the leucine-specific domain) and C-terminal residues 650–800 in the case of Tt LeuRS (Fig. 4b, black rectangles). Recent studies [49] suggest that these two domains require cooperative dynamics to recognize and bind the cognate tRNA and the observed anticorrelated motions could assist such tRNA binding event. Also, for Ec MetRS, EDA shows weaker correlated motion between a part of the aminoacylation domain (residues 250–400) and C-terminal residues 400–550 (Fig. 4b, squares). Similarly, it was also noted that for CEDA of Ef ProRS, the two protein segments (residues 125–250 and residues 375 to 475) maintain correlated movements, which appears to be quite weakened in the EDA-derived DCCM (Fig. 4c, rectangles). Taken together, these differences indicate that the coarse-grained CEDA tends to overestimate the extent of relative motion between residues, which could be due to the absence of local interactions, which were captured by all-atom simulation-derived EDA. Therefore, one would need to be cautious to estimate the extent of coupling between domain dynamics predicted by coarse-grained models.

#### EDA vs. NMA

Similar differences in the extent of correlations/anticorrelations were also observed in the comparative study of DCCMs between EDA and NMA, which are shown in the right-hand side matrices of Fig. 4, where the lower triangle corresponds to NMA-derived DCCM. The major contrasting regions are shown in the black squares/rectangles. In addition to those segments, a weak anti-correlated motion was observed between the CP and aminoacylation domains in EDA, when compared to the NMA (Fig. 4b, longer black rectangle).

### Coupling between local PBL dynamics and the global domain dynamics of ProRS

X-ray crystallographic study demonstrated a large-scale conformational change of the PBL upon substrate binding [3]. Molecular dynamics studies conducted recently in our group have demonstrated evidence of coupling between PBL and ED motions [4]. In the present study, an analysis of changes in the PBL dynamics and its impact on the ED motion was studied in the presence and absence of the substrate, prolyl-adenylate. The overall impact of the substrate binding on dynamics was evaluated by a) examining the changes in the thermal fluctuations of the backbone and b) by comparing the DCCM of substrate-bound and unbound ProRS systems.

### *B*-factors

**Fig. 5:**
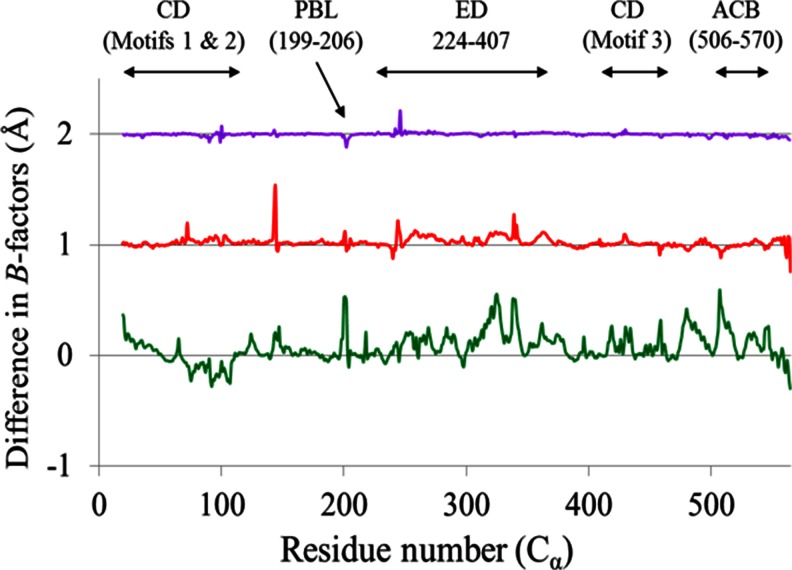
**a** The *B*-factors difference between the substrate-bound and unbound states of Ef ProRS. Color codes for the graphical sketches are *green*, EDA; *red*, CEDA; and *purple*, NMA

A plot of normalized *B*-factors of the C_α_ atoms of Ef ProRS is shown in Fig. 5, which revealed a difference in the backbone flexibility in various extents for the three methods. The EDA-derived *B*-factor analysis revealed a significant difference in the backbone flexibility patterns of the Ef ProRS in the presence of the substrate (Fig. 5). Especially, alteration in the flexibility of the ED and the PBL region was noticed upon substrate binding. In contrast, only minor differences resulted in the thermal fluctuations of Ef ProRS computed by the coarse-grained methods (NMA and CEDA) due to substrate-binding.

#### DCCMs

**Fig. 6:**
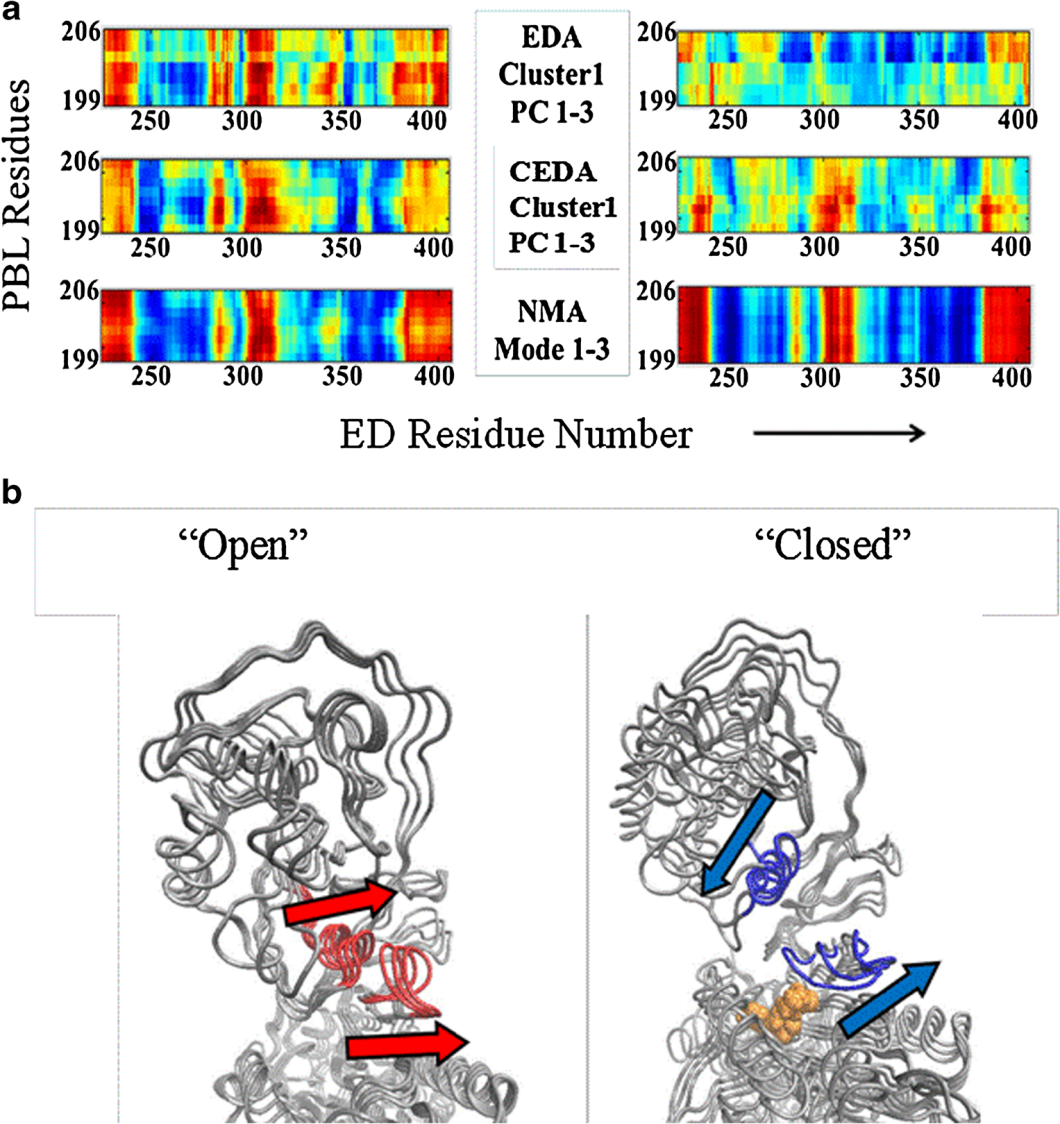
Dynamic cross-correlation map obtained from all-atom and coarse-grained simulations of Ef ProRS. **a** PBL (residues 199–206) vs. editing domain (residues 224–407) of substrate-unbound and bound Ef ProRS and **b** Cartoon representation showing the relative movements of the PBL (residues 199–206) vs. editing domain (residues 224–407) structural elements in substrate-unbound and bound states of Ef ProRS

The DCCM from EDA using all-atom MD trajectories revealed a prevalence of anticorrelated motion between the PBL and the ED in the presence of the substrate. In contrast, these two structural elements are found to be mainly engaged in correlated motion in substrate-unbound state (Fig. 6a). In particular, the secondary structural element consisting of beta-helix-turn-beta (residues 300–315, shown in red color in Fig. 6b) was found to be highly correlated with the PBL when the substrate was absent (“open” form in Fig. 6b). This secondary structural element, at the domain-domain interface, is in van der Waals contact with the PBL. There is significant alteration in the coupled dynamics of these two structural elements upon substrate binding. The “open” to “closed” conformational transition of the PBL due to substrate binding is expected to be favored by the existence of the anticorrelated motion between the PBL and the ED. This local dynamic coupling of these two structural elements was not identified in the analogous CEDA and NMA analyses (Fig. 6a) suggesting that substrate induced local changes are not captured by the coarse-grained models.

## Conclusions

Proteins exhibit complex internal motions. These intrinsic dynamics of proteins are believed to be related to their biological functions. Especially, the slow protein motions are suggested to be important for substrate binding and catalysis, as well as are more effective in propagating site-to-site signals (substrate-induced conformational changes) because of their cooperative and collective nature. However, the role of protein dynamics in enzyme catalysis is still hotly debated suggesting more studies, experimental as well as computational, are required. Recently, the computationally inexpensive coarse-grained simulations have emerged as invaluable tools for studying conformational changes in biomolecules. However, more studies are required to properly assess their ability to recognize local dynamics, as well as appreciate the coupling between global dynamics and local conformational changes.

In this study, an effort has been made to compare atomistic and coarse-grained simulations for studying the intrinsic dynamics of three AARSs. Our finding suggests that the global intrinsic dynamics can be predictable by atomistic and coarse-grained methods alike. The *B*-factor calculations revealed comparable thermal fluctuation of the protein backbone across all three simulation methods (Fig. 2). Furthermore, the overlap values for the conformational changes predicted by the coarse-grained and the atomistic simulations (averaged over lowest three modes) were high, varying between 0.77 and 0.86 (Table 1). The overlap of conformational changes of a specific domain was even higher (0.82–0.98) when compared between these methods (Table 2) indicating that the global dynamics in these modular proteins can be accurately studied using coarse-grained methods. This information is very helpful as the coarse-graining substantially reduces the computational cost, yet providing an insight of the collective motions involving large domains.

However, closer scrutiny of the relative domain motions simulated by various methods suggests that coarse-grained simulations provided rather imprecise information regarding the residue-residue fluctuations. This is revealed in the comparison of the relevant segments of the DCCMs (Fig. 4), which shows that coarse-grained simulations tend to overestimate the extent of the coupled motions in terms of intensity, presumably due to absence of the local interactions. In contrast, more detailed information was obtained from the MD-derived EDA.

In order to understand the effect of local changes on global dynamics, the change in the coupled dynamics due to the substrate binding for the Ec ProRS has been investigated. No significant difference in global dynamics was observed in both coarse-grained methods, although the starting conformation altered due to substrate binding. In contrast, spectacular changes were observed for the atomistic simulations (Fig. 5) of the substrate-bound and unbound Ec ProRS. Analysis of the ED and PBL indicates that their relative motions underwent a significant change due to substrate binding (Fig. 6a). The differences in thermal fluctuation arose due to changes in van der Waals interactions of the catalytically important PBL and ED (Fig. 6b), thus providing solid evidence that the substrate binding-induced changes are captured by only EDA, which is derived from all-atom MD simulations. This observed substrate-induced changes in the local interactions are first-ever to be modeled for prolyl tRNA synthetase and would serve as a key initiator of future efforts in exploring the global and local dynamics in this enzyme.

Taken together, these results suggest that the coarse-grained simulations are as effective as the all-atom simulations in providing a gross picture of the global collective dynamics involving modular proteins. These studies could provide valuable initial results using very small amounts of computational resources and efforts. However, they fail to capture details of the local changes, where all-atom MD simulations provide meaningful insights. Therefore, all-atom MD simulations should be a more reliable choice for studying local conformational alterations that trigger physico-chemical changes such as substrate-binding/product release or catalysis in these modular proteins.

## Electronic supplementary material

Below is the link to the electronic supplementary material.

ESM 1 (DOC 765 kb)

## Abbreviations

CMD: Coarse-grained molecular dynamics
CEDA: Coarse-grained essential dynamics analysis
CP: Connective polypeptide
Ec: *Escherichia coli*
ED: Editing domain
Ef: *Enterococcus faecalis*
EDA: Essential dynamics analysis
LeuRS: Leucyl-tRNA synthetase
MetRS: Methionyl-tRNA synthetase
MD: Molecular dynamics
NMA: Normal mode analysis
PCA: Principal component analysis
PBL: Proline-binding loop
ProRS: Prolyl-tRNA synthetase
Tt: *Thermus thermophilus*
