# Supplementary material for: Probing the global and local dynamics of aminoacyl-tRNA synthetases using all-atom and coarse-grained simulations
Source: J Mol Model. 2014 May 9;20(5):2245. doi: 10.1007/s00894-014-2245-1 (PMC4030129; doi:10.1007/s00894-014-2245-1)
Supplement: Supplementary file 1 — (DOC 765 kb) [file 894_2014_2245_MOESM1_ESM.doc]

**01/10/14**

**Probing the Global and Local Dynamics of Aminoacyl-tRNA Synthetases using All-atom and Coarse-grained Simulations**

Alexander M. Strom, Samuel C. Fehling, Sudeep Bhattacharyya* and Sanchita Hati*

Department of Chemistry, University of Wisconsin –Eau Claire, WI, 54702

To whom correspondence should be addressed: S.B.: phone: 715-836-2278; email: bhattas@uwec.edu; S.H.: phone: 715-836-3850; fax: 715-836-4979; email: [hatis@uwec.edu](mailto:hatis@uwec.edu);

Figure S1. RMSD of the C_α_ atoms from their initial coordinate as a function of time. Tt LeuRS (red), Ec MetRS (blue), Ef ProRS unbound state (green), Ef ProRS bound state (purple).


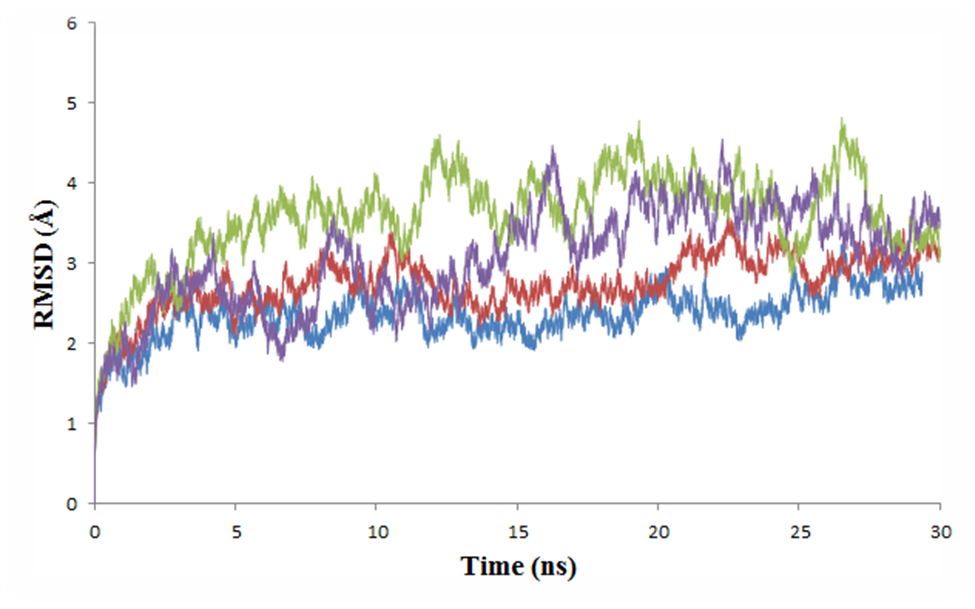


Figure S2. Secondary structure of the interfacial region between ED/CP and catalytic aminoacylation domains in a) Tt LeuRS, b) Ec MetRS, and c) Ef ProRS.
